# Mesothelial-to-Mesenchymal Transition Contributes to the Generation of Carcinoma-Associated Fibroblasts in Locally Advanced Primary Colorectal Carcinomas

**DOI:** 10.3390/cancers12020499

**Published:** 2020-02-21

**Authors:** Carlos H. Gordillo, Pilar Sandoval, Patricia Muñoz-Hernández, Lucía Pascual-Antón, Manuel López-Cabrera, José A. Jiménez-Heffernan

**Affiliations:** 1 Servicio de Anatomía Patológica, Hospital Universitario La Princesa, Instituto de Investigación Sanitaria Princesa (IP), 28006 Madrid, Spainpmunozhernandez@gmail.com (P.M.-H.); 2 Centro de Biología Molecular “Severo Ochoa”—CSIC, 28049 Madrid, Spain; pilarsandovalcorrea@hotmail.com (P.S.); lucia.pascual@cbm.csic.es (L.P.-A.)

**Keywords:** carcinoma-associated fibroblasts, mesothelial-to-mesenchymal transition, colorectal cancer, peritoneal metastasis

## Abstract

During peritoneal metastasis, cancer cells spread from abdominal solid tumors, disseminate through the peritoneal fluid and attach to and invade through mesothelial cells (MCs) that line the peritoneum. Intestinal adenocarcinomas originating in the mucosa infiltrate the submucosa, muscle layer, and serosa in order to finally colonize the peritoneal cavity. However, the mechanism by which metastatic cells leave the primary tumor and reach the peritoneal cavity has not been previously described. Hence, we investigate whether MCs lining visceral peritoneum, through a mesothelial-to-mesenchymal transition (MMT), are a source of carcinoma-associated fibroblasts (CAFs), which could contribute to cancer progression toward the peritoneal cavity. CAFs detected in biopsies from patients with superficially invasive colorectal cancer differed from locally advanced tumors. An aberrant accumulation of myofibroblasts expressing mesothelial markers was found in the stroma of deeply infiltrative tumors located in the neighborhood of a frequently activated mesothelium. We suggest that MMT is a key event in the early stages of peritoneal dissemination.

## 1. Introduction

Epithelial-to-mesenchymal transition (EMT) is a process by which differentiated epithelial cells undergo a deep molecular reprogramming and gradually acquire a mesenchymal phenotype characterized by elongated morphology, enhanced migratory and invasive capacity, and increased production of extracellular matrix (ECM) components. EMT participates in embryogenesis (type I EMT), wound healing, and organ fibrosis (type II EMT), as well as in metastasis (type III EMT) [1,2,3]. Type II EMT typically acts as a source of fibroblasts in order to reconstruct tissues following an injury. Likewise, organ fibrosis occurs when wound healing response persists, in part, due to a continual type II EMT process [4]. In previous studies, we demonstrated that a type II EMT, known as mesothelial-to-mesechymal transition (MMT), occurs after peritoneal damage. Myofibroblast conversion of mesothelial cells (MCs) contributes to peritoneal fibrosis associated with peritoneal dialysis and post-surgical peritoneal adhesions [5,6,7,8].

Malignant neoplastic tissue consists of transformed tumor cells surrounded by multiple non-cancerous cell populations, among which carcinoma-associated fibroblasts (CAFs) represent one of the major components of the stromal compartment. CAFs are activated fibroblasts essential in determining the tumor behavior in terms of proliferation, invasion, angiogenesis, and metastasis, through the diffusion of multiple growth factors, deposition of ECM elements, and/or the establishment of direct cell-to-cell contacts [9,10]. The origin of CAFs is under debate. Resident tissue fibroblasts, bone marrow-derived mesenchymal stem cells, circulating fibrocytes, epithelial cells (through type III EMT), and endothelial cells (through endothelial-to-mesenchymal transition) are all possible predecessors of CAFs [9,10,11,12,13]. However, MMT is considered as the principal origin of CAFs in the context of peritoneal metastasis [14]. Abdominal tumors, including gastric, colorectal, and ovarian cancers, frequently disseminate via a transcoelomic route [15]. Cancer cells floating into the peritoneal cavity invade the metastasizing peritoneal niche through a mainly transforming growth factor (TGF)-β1-mediated MMT induction, while a large proportion of cancers display mutational inactivation of the TGF-β pathway [16,17]. Accordingly, mesothelial-derived CAFs provide to the secondary peritoneal tumors of the adequate vascularization and ECM support to survive [17,18,19].

MMT-originated CAFs have been studied in peritoneal carcinomatosis [14], but little is known about their presence in primary neoplasms. MCs form a monolayer, known as mesothelium, that lines the pleura, peritoneum, and pericardium [15,20]. The mesothelium is composed of a parietal and a visceral surface. The latter delicately lines the outer surface of many organs, including most abdominal and thoracic ones [15]. Our hypothesis is that when abdominal neoplastic growth reaches the vicinity of the visceral mesothelial layer, it acts as an inducer of MMT. As a consequence, mesothelial-derived CAFs could facilitate tumor progression toward peritoneal cavity. To evaluate this hypothesis, we selected a malignant growth model by which neoplasms can be located at different distances from the visceral mesothelium. Intestinal adenocarcinomas originate in the mucosa and they progressively infiltrate the submucosa, muscle layer, and finally reach adipose tissue and mesothelium. If MMT contributes to the formation of CAFs, it would be observed in deeply infiltrative colorectal tumors located near the mesothelial layer.

## 2. Results 

### 2.1. Deeply Invasive Colon Carcinomas Show Numerous Carcinoma-Associated Fibroblasts (CAFs) Expressing Mesothelial Cell Markers

**Figure 1:**
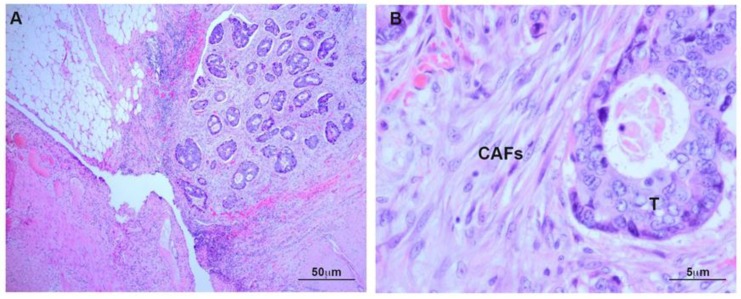
Deeply invasive colon carcinoma. (**A**) Haematoxylin-Eosin (H&E) staining of malignant neoplastic glands invading the adipose tissue and reaching the visceral peritoneal layer; (**B**) At higher magnification, CAFs surround a tumor gland. T: Tumor; CAFs: Carcinoma-associated fibroblasts.

**Figure 2:**
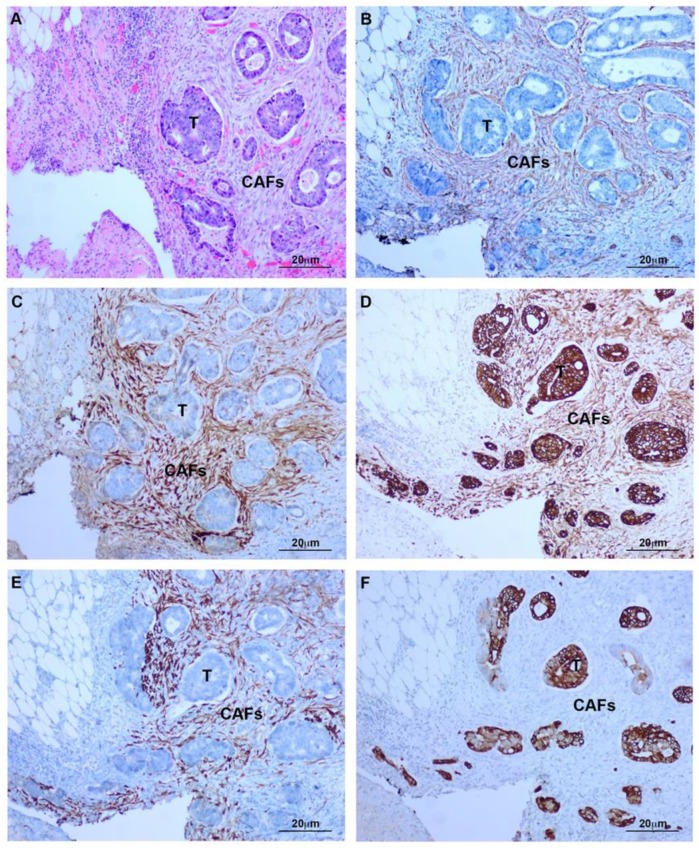
MMT is frequently observed in deeply invasive colon carcinomas. Serial sections from the same area. (**A**) Tumoral glands and stroma at the serosa surface (H&E); (**B**) Stromal myofibroblasts showing α-SMA expression; (**C**) Calretinin expression is present in CAFs and absent in tumor cells; (**D**) Pancytokeratin is expressed by neoplastic cells and myofibroblasts; (**E**) CK7 expression is limited to CAFs; (**F**) CK20 staining is limited to neoplastic cells. T: Tumor; CAFs: Carcinoma-associated fibroblasts.

**Figure 3:**
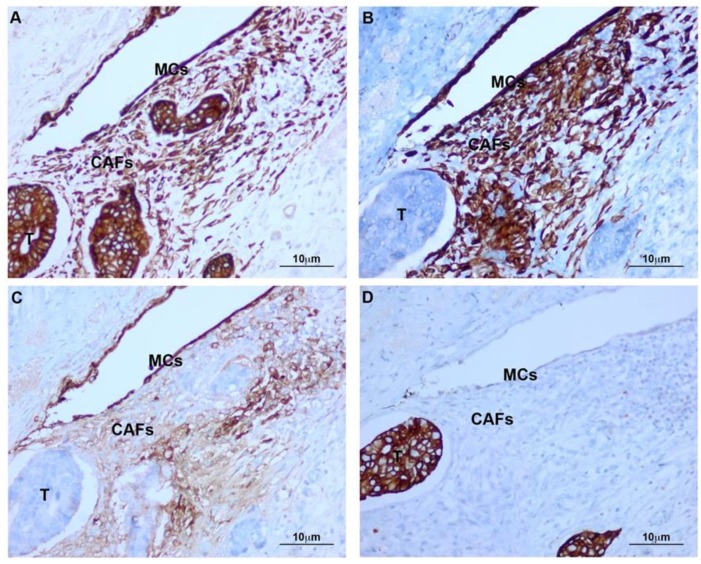
Infiltrative neoplastic glands reach a well-preserved mesothelial layer. Serial sections from the same area. (**A**) Pancytokeratin is expressed by mesothelium, stromal fibroblasts, and neoplastic glands; (**B**) CK7 is evident in the mesothelium and stromal fibroblasts, but absent in tumoral cells; (**C**) Mesothelin is expressed by MCs and stromal fibroblasts, while neoplastic cells are negative; (**D**) CK20 is present in tumor cells and absent in MCs and myofibroblasts. T: Tumor; CAFs: Carcinoma-associated fibroblasts; MCs: Mesothelial cells.

**Figure 4:**
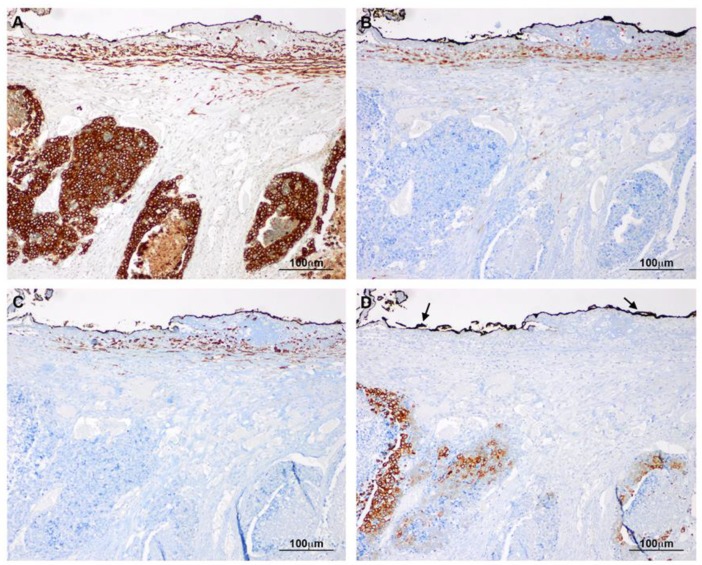
Gradient of expression of mesothelial markers between the deepest and most superficial CAFs. Serial sections from the same area. (**A**) Tumoral glands and CAFs located near the visceral mesothelial layer (submesothelium) show pancytokeratin expression; (**B**) Expression of calretinin is limited to CAFs of submesothelial location; (**C**) Similarly, CK7 expression is limited to submesothelial CAFs. Tumoral glands show no CK7 expression; (**D**) As opposed to CK7, CK20 is expressed by tumor cells and not by CAFs. Arrows point to the presence of ink routinely used during sample processing for limit recognition during microscopic analysis.

**Figure 5:**
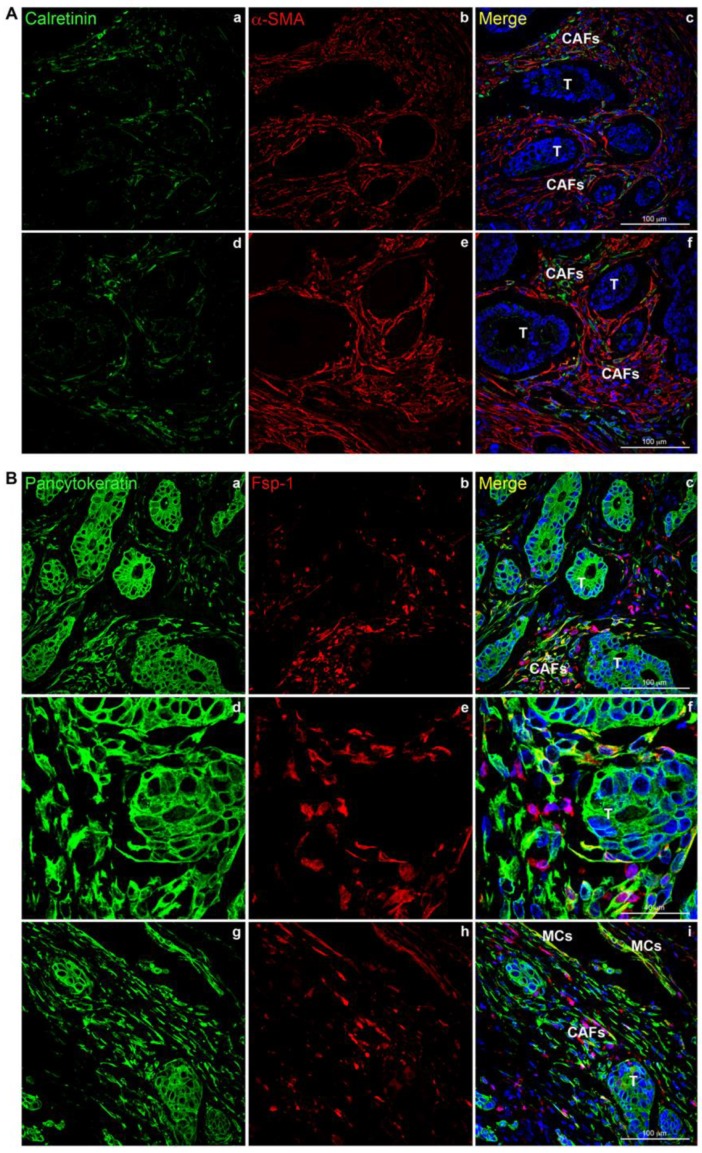
Mesothelial-derived CAFs co-express mesothelial and mesenchymal markers in colon carcinomas reaching the serosa layer. (**A**) Representative stromal areas surrounding deeply invasive cancer nodules show overlapped staining for calretinin and α-SMA (**a**–**f**). (**B**) CAFs co-expressing pancytokeratin and Fsp-1 are adjacent to pancytokeratin-positive tumor glands (**a**–**c**). At higher magnification, Fsp-1 positive fibroblasts closely surround a deep tumor nodule (**d**–**f**). Activated MCs forming a monolayer co-express pancytokeratin and Fsp-1 (**g**–**i**). T: Tumor; CAFs: Carcinoma-associated fibroblasts; MCs: Mesothelial cells.

Histology of deeply invasive tumors showed neoplastic glands with associated reactive stroma containing numerous CAFs (Figure 1). CAFs showed fibroblastic morphology and were embedded in an ECM with a variable number of lymphocytes and macrophages. Tumor glands and stroma were in close relation with MCs from the peritoneal visceral layer (Figure 2). Immunostaining of serial sections showed overlapped expression areas of the CAF marker alpha-smooth muscle actin (α-SMA) and mesothelial markers, such as calretinin, pancytokeratin, and cytokeratin 7 (CK7) (Figure 2 and Figure 3). To a less extent, expression of wilms tumor-1 (WT1) and mesothelin was also detected in areas bearing CAFs (Figure 3). The co-expression of mesothelial and mesenchymal markers in CAFs supports the notion that they derive from MCs via a MMT process. As expected, neoplastic cells also showed expression of pancytokeratin. An interesting finding was that CAFs expressed cytokeratin 7 (CK7), but not cytokeratin 20 (CK20) (Figure 2 and Figure 3). It is well known that MCs express CK7 but not CK20 and, conversely colon carcinoma cells express CK20 but not CK7. The cytokeratin expression pattern seen in CAFs (CK7+CK20−) argues against a potential origin from neoplastic cells via EMT [18,21,22]. Similarly, a gradient of expression of mesothelial markers from the superficial layer to the deepest zone of locally advanced colon carcinomas was observed. MC-derived CAFs were mainly accumulated in the vicinity of the visceral mesothelial layer (Figure 4). In a control visceral peritoneum, there was no expression of α-SMA other than that in the smooth muscle cells of the vessel walls and as expected, the staining for mesothelial markers, including CK7, calretinin, mesothelin and WT1 was limited to the preserved mesothelium (Figure S1). Accordingly, dual-immunofluorescence staining confirmed the accumulation of MC-derived CAFs in the tumor surrounding stroma, where typical mesothelial (calretinin) and myofibroblast (α-SMA) markers overlapped. The presence of pancytokeratin-positive colon tumor nodules reaching the mesothelial monolayer was frequently observed. At this zone, pancytokeratin-positive MCs lining the visceral peritoneum begin to express fibroblast-specific protein 1 (Fsp-1), indicating to be in an activated stage of the MMT. The submesothelial localization of MCs negative for mesenchymal markers suggested to be in a transitional MMT stage. On the contrary, the presence of CAFs negative for mesothelial markers depicts that the MMT has been completed (Figure 5).

### 2.2. CAFs From Superficially Invasive Colon Carcinomas Do Not Express Mesothelial Cell Markers

**Figure 6:**
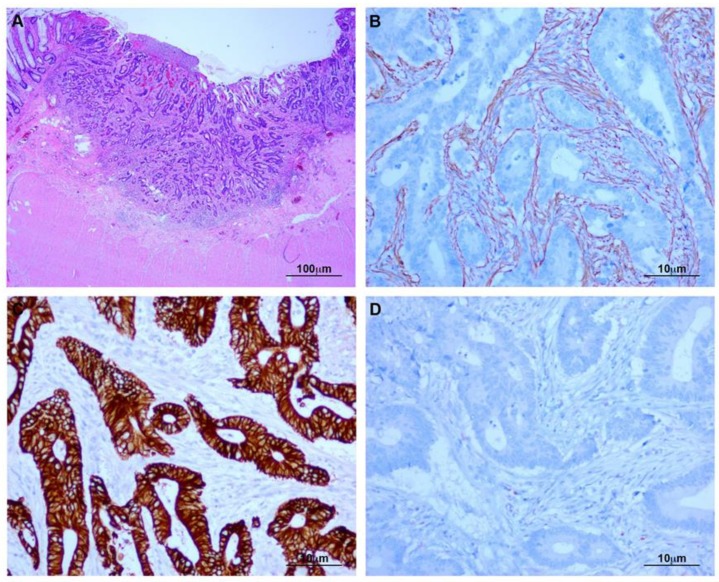
Superficially invasive colon carcinoma. (**A**) H&E staining of a superficially invasive intestinal adenocarcinoma with neoplastic growth limited to the submucosa; (**B**) CAFs show expression of α-SMA; (**C**) Pancytokeratin is expressed by cancer cells and absent in fibroblasts; (**D**) Calretinin-negative myofibroblasts and tumor cells.

Superficially invasive colon cancers showed neoplastic cells adjacent to a reactive stroma containing α-SMA-positive CAFs. Tumors were located far from the visceral peritoneal layer. Indeed, a wide and uninvolved muscle layer separated them. In all cases, CAFs failed to express MC markers. Pancytokeratin and calretinin were not detected in stromal areas surrounding tumors. As expected, tumor cells were positive for typical colon cancer markers, including pancytokeratin (Figure 6).

## 3. Discussion

The role of EMT in cancer progression has focused mainly on the mesenchymal conversion of neoplastic cells [2,23]. This type of EMT is known as type III and drives neoplastic cells to acquire a mesenchymal-like phenotype allowing metastatic progression [1,2,23]. In a previous study, we revealed, for the first time, that type II EMT, the one implicated in tissue fibrosis, can also contribute to metastasizing of carcinomas to secondary organs [18]. Interestingly, the treatment of MCs with conditioned media from ovarian or colorectal carcinoma cell cultures has resulted in phenotype and molecular changes reminiscent of MMT [18]. Therefore, in peritoneal carcinomatosis, secretome of abdominal tumors that have reached the peritoneal cavity modificates the mesothelium in order to be colonized via a MMT process. Consequently, mesothelial-derived CAFs are accumulated in the underlying mesothelial zone, rendering the peritoneal niche susceptible for implantation, invasion, and growth of secondary tumors [14,17,18]. In these studies, in vitro and ex vivo experiments were combined with a mouse model of peritoneal metastasis in order to obtain a whole picture of the peritoneal carcinomatosis process. However, the identification in biopsies from patients with peritoneal metastasis of myofibroblasts expressing mesothelial markers in the proximity of carcinoma implants constituted the most reliable proof that a MMT process takes place [17,18]. Here we show, for the first time, that CAFs found in locally advanced primary abdominal neoplasms arise from the mesenchymal conversion of MCs lining the visceral serosa. Evidence of MMT was observed in deeply invasive colon carcinomas in which tumor growth is located close to or invades the mesothelium (stage T3 and T4, respectively). Serial sections of advanced invasive neoplasms showed abundant α-SMA-positive spindle-like cells embedded in the reactive stroma tissue surrounding tumor nodules, overlapping with areas with marked immunohistochemical staining for mesothelial markers. More precisely, mesothelial-derived CAFs were easily identified through dual-immunofluorencence staining for mesenchymal (α-SMA and Fsp-1) and mesothelial markers (calretinin and pancytokeratin). Cytokeratins are intermediate filaments that form part of the cytoskeleton of epithelial cells and are absent in mesenchymal cells such as fibroblasts. During MMT, MCs progressively lose their epithelial characteristics, in part, through the downregulation of a broad spectrum of cytokeratins [5]. This fact leads us to speculate that we could be underestimating the real population of CAFs with mesothelial origin. The colocalization of cytokeratins and mesenchymal markers in cells showing fibroblastic morphology and location can be considered as evidence of partial MMT. Similarly, submesothelial localization of MCs with spindle-like morphology but negative for mesenchymal markers can represent transitional stages, where despite MCs having acquired invasive properties, the conversion is unfinished. Once MMT is completed, the total loss of mesothelial markers makes it impossible to know the real origin of CAFs. That is why some authors still debate as to whether type II EMT really occurs in vivo [24]. On the other hand, a specific cytokeratin expression profile allowed separate identification of mesothelial-derived CAFs from tumor cells [18,21,22]. In this regard, intestinal carcinoma cells expressed CK20 but not CK7 and, conversely, MCs expressed CK7 but not CK20. This result strongly supports the mesothelial origin of CAFs and definitely excludes the mesenchymal conversion of neoplastic cells via a type III EMT process. In addition to superficial MCs, deeply invasive colon carcinoma biopsies revealed a relevant subset of calretinin-positive CAFs. Calretinin is a calcium-binding protein expressed by MCs and mesothelioma, neurons, adipocytes, and mast cells and is absent in resident fibroblasts [25,26]. Expression of other mesothelial markers, such as mesothelin and WT1, was also observed in CAFs surrounding infiltrative tumors located in proximity of the visceral mesothelium. For these molecules, the immunostaining was less intense than that observed for cytokeratins and calretinin. Similar expression of mesothelial markers by subserosal fibroblasts, including CAFs, has been reported in a few other studies where pathologists are adviced not to confuse cytokeratin-positive fibroblasts for neoplastic cells [27,28,29]. In addition to cytokeratins, Chen J.H. and Borges M. [29] describe the subserosal expression of other mesothelial markers as well. For these authors, such expression is evident, but it is not interpreted as a MMT process. It was thought to represent evidence of a reparative mechanism by which subserosal progenitor cells differentiate into MCs. However, our data strongly disagree with this theory, since we have frequently found Fsp-1 (fibroblast-specific protein) in the preserved mesothelium next to advanced neoplasm regions. Focal expression of mesenchymal markers in superficial MCs was previously denoted in other MMT-related pathologies [8]. This situation indicates an early stage of the MMT process. Interestingly, Fsp-1 has been reported to be mainly confined to transitioning fibroblasts, while α-SMA predominates in, but is not limited to, morphologically and functionally mature myofibroblasts, this being the reason why in the deepest submesothelial zones CAFs expressing α-SMA are more abundant than those positive for Fsp-1 [30]. Alternatively, our study supports the role of MMT in the formation of CAFs, since the expression of mesothelial markers was absent in the reactive stroma adjacent to early invasive colonic carcinomas. In this case, tumors showed no relation to the visceral mesothelium, since a wide muscle layer separated mucosa from serosa. Similarly, no expression of typical mesothelial markers was previously observed in CAFs from breast and cutaneous carcinomas, both being locations devoid of mesothelial participation [18]. The mesothelial membrane forms the lining of body cavities, encasing the heart, the pleural cavity, the peritoneum, and the female and male internal reproductive organs. Interestingly, previous studies led to the evidence that the parietal layer shows morphological, biochemical, and metabolic differences with visceral MCs lining internal organs [31,32]. In terms of MMT, we have not observed differences between both coelomic layers. MMT affecting the visceral mesothelium seems to be in accordance with previous studies from our group showing a similar accumulation of meothelial-derived CAFs in the parietal peritoneum metastasized by ovarian, endometrial, or colorectal cancers [17,18].

MMT is a step-wise process that requires a profound molecular reprogramming [7]. Over the last decade, numerous studies have contributed to unraveling the complex network of molecules that govern the MMT process with a unique purpose consisting of the design of therapeutic strategies to prevent or reverse the MMT itself, or alternatively treat its effects, such as cellular invasion, ECM accumulation, or angiogenesis [33,34]. In the context of peritoneal carcinomatosis, increasing studies point to MMT as a therapeutic target to restrict the implantation of secondary tumors in the peritoneum [17]. However, peritoneal carcinomatosis defines an advanced stage of disease difficult to control. Therefore, we propose visceral-related MMT as a therapeutic target to prevent the spread of secondary peritoneal tumors.

## 4. Materials and Methods

### 4.1. Patients 

This is a retrospective study performed on surgical specimens corresponding to intestinal resections from patients with large bowel carcinoma. Informed written consent to use surgical samples was obtained from the patients with the approval of the Ethics Committee of Hospital Universitario Puerta de Hierro Majadahonda (Madrid, Spain, ethic approval number: 11.17). The pathologic diagnosis was performed following the protocol for the examination of specimens from patients with primary carcinoma of the colon and rectum, proposed by the College of American Pathologists (version: colon rectum 4.0.0.0). Two groups of patients with intestinal adenocarcinoma were considered. The first group corresponded to a total of 16 surgical specimens showing deeply invasive carcinomas in which the neoplastic growth almost reached the visceral peritoneum (pT3) or invaded it (pT4). Ten of them showed a pT3 level of invasion, which means that the tumor has invaded the muscle layer of the intestinal wall and extended into the surrounding adipose tissue, but without infiltrating the visceral peritoneum. Six cases showed a pT4 level that meant that the outer layer of the intestinal wall, the visceral peritoneum, has been invaded. For comparison, a second group included a total of 15 surgical specimens showing carcinomas with superficial invasion. In these cases, the tumor growth was limited to the submucosa with no muscle layer or peritoneal involvement. All samples were fixed in neutral-buffered 3.7% formalin and embedded in paraffin to obtain serial sections 3 μm thick. After the routine pathologic study, relevant tissue samples were selected for immunohistochemical and immunofluorescence analysis.

### 4.2. Immunohistochemical Analysis

Immunohitochemistry was performed using an automated immunostainer (Ventana Benchmark Ultra, Ventana Medical Systems, Arizona, USA). The following primary antibodies (Ventana Medical Systems) were used: α-SMA, pancytokeratin (AE1/AE3), CK7, CK20, calretinin, mesothelin, and WT1. Antibodies were visualized using 3,3’-diaminobenzidine (DAB) (Ventana Medical Systems) as chromogen, and tissue sections were finally counterstained with haematoxylin.

### 4.3. Dual-Immunofluoresecence Analysis

Dual-immunofluorescence staining was performed on formalin fixed paraffin embedded tissues using antibodies to visualize calretinin (Abcam, Cambridge, UK) and α-SMA (Sigma-Aldrich, St Louis, MO, USA) or pancytokeratin (clone PCK-26; Sigma-Aldrich) and Fsp-1 (Dako, Glostrup, Denmark). The sections were heated to expose the hidden antigens using Real Target Retrieval Solution containing citrate buffer, pH 6.0 (Dako). Secondary antibodies Alexa Fluor 647 and Alexa Fluor 555 (Thermofisher Scientific, Massachusetts, USA) were incubated at room temperature. The nucleus was stained with 4,6-diamidino-2-phenylindole (DAPI) (Thermofisher Scientific). Finally, the preparations were visualized with a LSM710 confocal microscope (Zeiss, Oberkochen, Germany). Negative controls omitting primary antibodies did not give rise to any detectable labelling.

## 5. Conclusions

This study reveals, for the first time, the presence of MMT-derived CAFs in primary colorectal tumors. Mesothelial-derived CAFs could contribute to cancer progression in locally advanced stages. As a consequence, visceral mesothelial layer disruption could facilitate the access of the tumor into the peritoneal cavity. There is an opportunity to prevent peritoneal dissemination of primary abdominal tumors diagnosed at early stages, since MMT is a modulable process.

## Supplementary Materials

The following are available online at https://www.mdpi.com/2072-6694/12/2/499/s1, Figure S1: Control visceral peritoneum.
