# Supplementary material for: Mesothelial-to-Mesenchymal Transition Contributes to the Generation of Carcinoma-Associated Fibroblasts in Locally Advanced Primary Colorectal Carcinomas
Source: Cancers (Basel). 2020 Feb 21;12(2):499. doi: 10.3390/cancers12020499 (PMC7072259; doi:10.3390/cancers12020499)
Supplement: Supplementary file 1 [file cancers-12-00499-s001.pdf]

cancers

MDPI

Communication

Mesothelial-to-Mesenchymal Transition Contributes to the Generation of Carcinoma-Associated Fibroblasts in Locally Advanced Primary Colorectal Carcinomas

Carlos H. Gordillo [1,†], Pilar Sandoval [2,†], Patricia Muñoz-Hernández [1], Lucía Pascual-Antón [2], Manuel López-Cabrera [2,*,‡] and José A. Jiménez-Heffernan [1,*,‡]

Supplementary Material

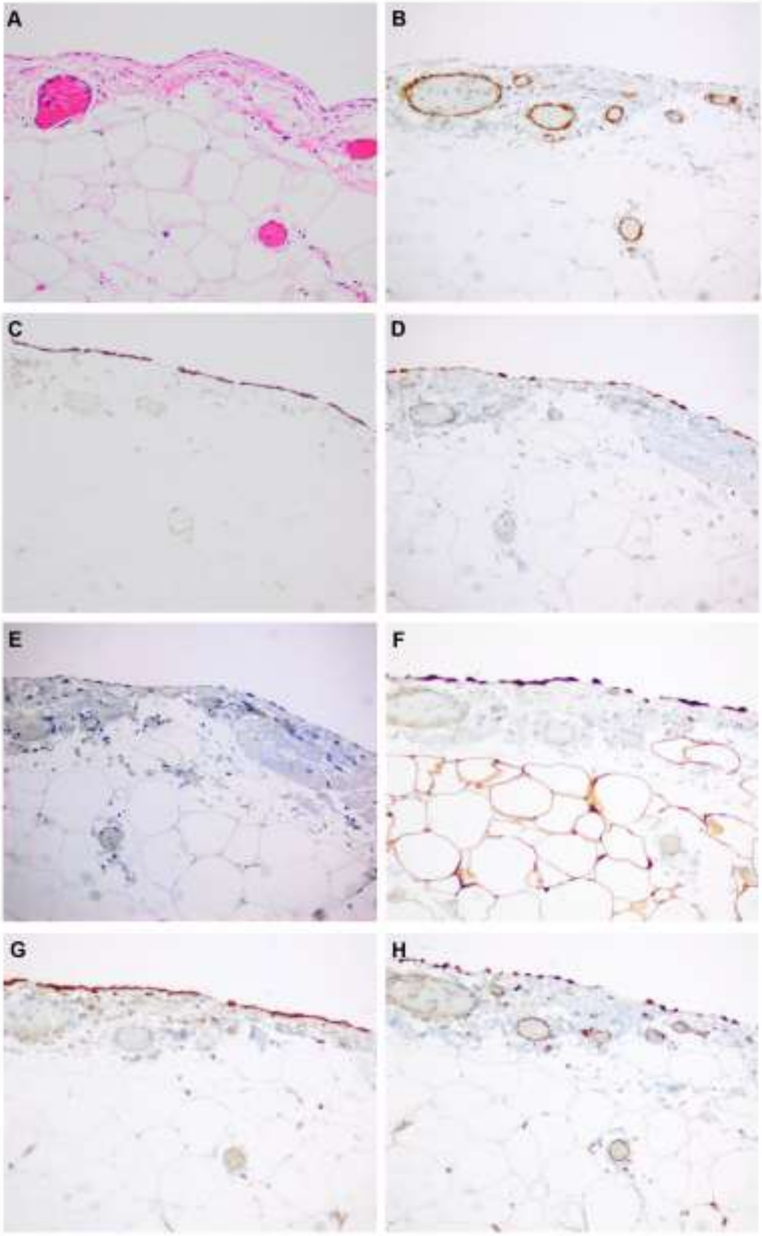

Figure 1. Control visceral peritoneum.
